# Effect of Functional Electrical Stimulation in Convalescent Stroke Patients: A Multicenter, Randomized Controlled Trial

**DOI:** 10.3390/jcm12072638

**Published:** 2023-04-01

**Authors:** Shuji Matsumoto, Megumi Shimodozono, Tomokazu Noma, Kodai Miyara, Tetsuya Onoda, Rina Ijichi, Takashi Shigematsu, Akira Satone, Hidenobu Okuma, Makiko Seto, Masanori Taketsuna, Hideaki Kaneda, Miyuki Matsuo, Shinsuke Kojima, the RALLY Trial Investigators

**Affiliations:** 1 Center of Medical Education, Faculty of Health Sciences, Ryotokuji University, Chiba 279-8567, Japan; 2 Department of Rehabilitation and Physical Medicine, Mito Clinical Education and Training Center, University of Tsukuba Hospital, Mito 310-0015, Japan; 3 Department of Rehabilitation and Physical Medicine, Graduate School of Medical and Dental Sciences, Kagoshima University, Kagoshima 890-8544, Japan; 4 Department of Rehabilitation, Faculty of Health Science, Nihon Fukushi University, Aichi 470-3295, Japan; 5 Department of Rehabilitation, Kagoshima University Hospital, Kagoshima 890-0075, Japan; 6 Department of Rehabilitation, Kirishima Medical Center, Kagoshima 899-5112, Japan; 7 Department of Rehabilitation, Kirishima Sugiyasu Hospital, Kagoshima 899-4201, Japan; 8 Department of Rehabilitation, Hamamatsu City Rehabilitation Hospital, Shizuoka 433-8511, Japan; 9 Department of Rehabilitation, Tokachi Rehabilitation Center, Hokkaido 080-0835, Japan; 10 Department of Rehabilitation, Kumamoto Takumadai Rehabilitation Hospital, Kumamoto 862-0924, Japan; 11 Department of Rehabilitation, Nagasaki Kita Hospital, Nagasaki 851-2103, Japan; 12 Translational Research Center for Medical Innovation, Kobe 650-0047, Japan

**Keywords:** electrical stimulation, gait, stroke, lower extremity, walking

## Abstract

Background: We evaluated whether the Walkaide^®^ device could effectively improve walking ability and lower extremity function in post-stroke patients with foot drop. Patients aged 20–85 years with an initial stroke within ≤6 months and a functional ambulation classification score of 3 or 4 were eligible. Materials and Methods: Patients were randomly allocated to the functional electrical stimulation (FES) or control group at a 1:1 ratio. A 40 min training program using Walkaide was additionally performed by the FES group five times per week for 8 weeks. The control group received the 40 min training program without FES. Results: A total of 203 patients were allocated to the FES (*n* = 102) or control (*n* = 101) groups. Patients who did not receive the intervention or whose data were unavailable were excluded. Finally, the primary outcome data of 184 patients (*n* = 92 in each group) were analyzed. The mean change in the maximum distance during the 6-MWT (primary outcome) was 68.37 ± 62.42 m and 57.50 ± 68.17 m in the FES and control groups (difference: 10.86 m; 95% confidence interval: −8.26 to 29.98, *p* = 0.26), respectively. Conclusions: In Japanese post-stroke patients with foot drop, FES did not significantly improve the 6 min walk distance during the convalescent phase. The trial was registered at UMIN000020604.

## 1. Introduction

While age-standardized rates of stroke mortality have decreased worldwide in the past two decades, both the absolute number of people experiencing a stroke every year and the number of stroke survivors have been increasing [1]. Stroke survivors, often with disabilities, cannot actively dorsiflex the foot during the swing phase of gait (known as foot drop). Foot drop is a common disorder following stroke and is associated with severe motor impairment, weakness or lack of voluntary control of the dorsiflexor muscles of the ankle joint, and increased spasticity of the plantar flexor muscles [2]. Foot drop is classified as the inability to dorsiflex the foot and is most commonly caused by weakness of the dorsiflexors (and abductor muscles) and/or overactivity of the plantar flexor muscle group (and adductor muscles) [3]. Foot drop decreases gait velocity and limits functional mobility. Traditionally, an ankle-foot orthosis (AFO) is used for foot drop [4]; however, the effects of the AFO attachment include an increase in walking speed and stride [5].

Functional electrical stimulation (FES)—an alternative to foot drop treatment—is designed to restore motor function in paralyzed limbs by electrically stimulating the neuromuscular system during ambulation [6]. Several randomized trials have demonstrated that FES devices have similar benefits as AFOs for key walking measures in patients with foot drop caused by stroke [7,8,9,10]. However, only a few studies have exclusively focused on convalescent stroke patients (≤6 months post-stroke). Furthermore, most studies of FES were conducted in Europe and the U.S., where lifestyles are very different from those in Japan and shoes are worn even indoors [11,12,13]. In Japan, people do not usually wear shoes indoors. Hence, whether FES devices would be effective for Japanese convalescent stroke patients with foot drop remains unclear.

Walkaide^®^ (Innovative Neurotronics, Reno, NV, USA) is an FES device suitable for walking with bare feet as it has a tilt sensor [8]. The Walkaide^®^ FES system is a self-contained FES device with built-in tilt sensor that attaches with a cuff to the leg below the knee. When the leg is tilted back at the end of stance, stimulation of the common peroneal nerve is initiated, producing dorsiflexion of the ankle to facilitate leg clearance during swing. When the leg is tilted forward at the end of the swing phase, stimulation is terminated. In this trial, we examined whether the Walkaide^®^ FES system effectively improves walking ability and lower extremity function in Japanese patients with unstable gait from foot drop post-stroke.

## 2. Materials and Methods

### 2.1. Study Design

We performed a randomized, controlled, open-label trial enrolling patients with post-stroke hemiplegic gait disorder (foot drop) from 30 rehabilitation centers across Japan (study sites and trial investigators are provided in Appendix B). The study protocol was approved by the ethics committees of all participating institutions. This study was conducted in accordance with the tenets of the Declaration of Helsinki. A detailed description of the study design and the methods has been published previously, with a brief summary provided here [14]. The trial was registered with ClinicalTrials.gov, registration number was NCT02898168 (https://clinicaltrials.gov/ct2/show/NCT02898168) (accessed on 22 March 2023).

### 2.2. Participants

Patients aged 20–85 years were eligible for inclusion if they had an initial stroke within ≤6 months with a functional ambulation classification (FAC) score of 3 or 4 (FAC is a scale of 0–5, where 3 indicates supervision or standby guarding and 4 indicates independent on level surfaces) prior to providing consent for this study [15]. Patients who could not complete the rehabilitation program due to comorbidities (including severe osteoarthritis, liver, kidney, or cardiovascular dysfunction) were excluded. In addition, the exclusion criteria included the following: contraindication to the device (e.g., metallic implant, implanted medical electrical device, past or current epilepsy, and uncontrolled seizure disorder); neuromuscular disorders (excluding stroke); mental disorder; severe edema of a lower extremity; evidence of deep venous thrombosis or thromboembolism; severe atherosclerosis of the lower extremities; or musculoskeletal systems that would potentially affect gait; and a high risk of falling. Patients with other conditions that may affect the outcome were also excluded (e.g., use of FES or a robot suit within 1 month, botulinum toxin injections or phenol nerve block injection within 6 months, or severe sensory dysfunction or higher brain dysfunction before consenting to this study).

After the enrolled patients provided written informed consent, the treating physician and physical therapists evaluated FES compatibility for a screening period of up to 7 days. Patients were excluded if any of the following was observed: (1) unresponsiveness to the FES device, (2) intolerance to continuous stimulation, and (3) gait function improved significantly during the screening period. The full eligibility criteria are provided in the previous paper [14].

### 2.3. Randomization and Masking

After the screening period, the enrolled patients were randomly allocated to either the FES or control group (1:1 ratio) with a minimization method using an electronic data capturing system—eClinical Base (Translational Research Center for Medical Innovation) (https://www.tri-kobe.org/support/tools/, accessed on 22 March 2023). The allocation was centralized using web-based randomization software (eClinical Base). Randomization was stratified according to the following factors: FAC score 3 or 4, age < 65 years, type of stroke (ischemic or hemorrhagic), and institution.

This was an open-label trial with both patients and physicians unblinded to the treatment allocation. However, all outcomes except the examinations with FES and questionnaires were evaluated by investigators blinded to the treatment allocation. The 10 m walk tests (10-MWT) were videotaped, and gait disturbance was evaluated by an independent central adjudication committee [14].

### 2.4. Procedures

A 60 min usual physiotherapy treatment was provided to both the FES and control groups 5 days a week over 8 weeks (40 days), consisting of basic activity training as follows: (1) mat exercise, (2) standing up and sitting down, (3) ambulation with assistive devices or manual support, and/or range of motion (ROM) training, and/or gait training using an AFO (if the patient had already used it at the time of recruitment). In addition to the usual rehabilitation training, the patients included in this study received their allocated program (FES or control). Any rehabilitation programs initiated before this trial were continued under the condition that the intervals, duration, or contents remained the same throughout the trial [14]. The study participants received the allocated program (FES or control) in addition to the usual training. To ensure homogeneity of treatment at the 30 facilities, an educational program was implemented in advance

#### 2.4.1. FES Group

The participants in the FES group underwent a 40 min training program 5 days a week for 8 weeks with Walkaide^®^ (Teijin Pharma Ltd., Tokyo, Japan) (the stimulation parameters have been detailed previously [14]). WalkAide electrical stimulation was performed by applying electrodes to the peroneal nerve bifurcation and the tibialis anterior muscle using an asymmetrical biphasic pulse. Stimulation was performed at voltages ranging from 121 V at 1 KΩ to 150 V at 1 MΩ, and the electrodes were fixed at the voltage at which appropriate ankle dorsiflexion for walking was obtained. After appropriate dorsiflexion was obtained, the pulse width (25–300 μs) and stimulation period (maximum 3 s) were adjusted to set the appropriate stimulation pattern for each subject [14]. The therapeutic electrical stimulation (TES) mode was used in patients with an FAC score of 3. In contrast, the HAND mode (manual electrical stimulation) and TILT mode (electrical stimulation delivered in the swing phase based on a tilt sensor) were used in patients with an FAC score of 4. Treatment modes were selected to be adjusted by qualified program providers. The use of AFO was prohibited during FES training. All training was overviewed by physicians or physical therapists.

#### 2.4.2. Control Group

A 40 min training program without FES was provided 5 days a week for 8 weeks. For patients with an FAC score of 3, self-stretching and foot dorsiflexion ROM training (triple foot triceps stretch training to extend the foot dorsiflexion ROM) was added; for patients with an FAC score of 4, gait training using AFO was added.

### 2.5. Outcomes

The primary outcome was the mean change in the distance covered during the 6 min walk test (6-MWT), defined as the difference in the distances (meters) during a 6-MWT performed barefoot at week 0 (pretreatment period) and until week 8 [16]. The secondary outcomes included the changes in the 10-MWT [17,18], performed at a comfortable walking speed, of which the average value of two measurements was calculated [16,19,20]. Other secondary outcome measures included were as follows: (1) the 6-MWT with an AFO or FES; (2) the Fugl–Meyer assessment score; (3) the modified Ashworth scale score; (4) the active and passive ROM for ankle dorsiflexion; (5) the Timed Up and Go test; (6) Stroke Impact Scale score; (7) patient-reported outcome measures (questionnaire); and (8) gait evaluation by the care providers (videotaped). All outcomes were collected during week 0 and week 8. For the safety assessment, any adverse events (AEs) were collected regardless of their severity.

### 2.6. Statistical Analysis

The planned study population comprised 200 patients (100 in each group). With 200 patients, a difference of 43.8 m in the 6-MWT was detectable with a statistical power of 80%, based on the assumption that the standard deviation (SD) was 110 m. Two-sample t-tests were performed to compare the change in the 6-MWT distance between the groups. All analyses were predefined in the statistical analysis plan before locking the database and were conducted using SAS version 9.4 (SAS Institute Inc., Cary, NC, USA). Data are expressed as means with SDs for continuous variables and frequencies and percentages for discrete variables unless specifically mentioned. The significance level was set at *p* < 0.05 (two-tailed). As the primary analysis was conducted in accordance with the modified intention-to-treat principle, the analysis excluded patients whose intervention was not initiated, but included patients whose intervention was prematurely discontinued, as prespecified in the protocol.

## 3. Results

**Figure 1:**
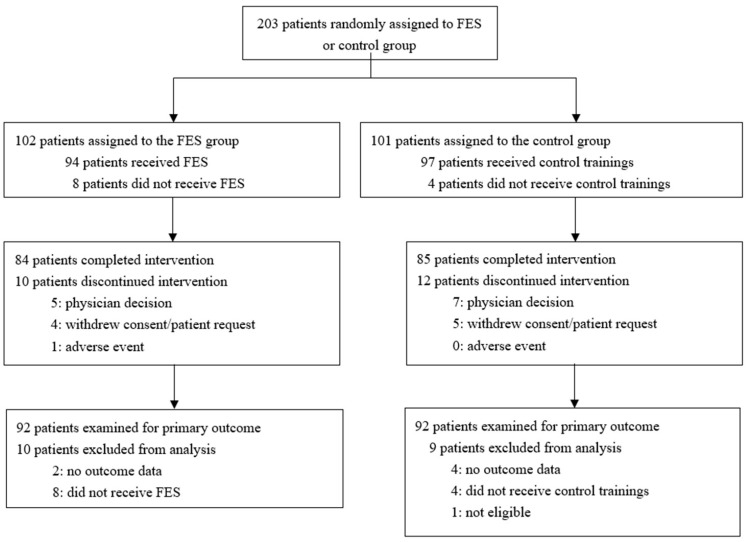
Trial profile. Abbreviation: FES, functional electrical stimulation.

The study was conducted with enrolment from May 2016 to December 2018. A total of 203 patients were randomly assigned to the FES (*n* = 102) or control group (*n* = 101) (Figure 1). Eighty-four patients in the FES group and 85 patients in the control group completed the intervention. After excluding 19 patients who did not receive the intervention or whose data were not available, the data of the primary outcomes of 184 patients (92 in each group) were analyzed. The baseline characteristics of the two groups were similar (Table 1). The mean age at recruitment was 64 (SD: 11) years. A total of 138 (75%) patients were men. A total of 102 patients (55%) had cerebral infarction, while 101 patients (55%) had an FAC score of 3.

The primary outcome (mean change in the maximum distance during the 6-MWT (barefoot) from the baseline to the end of the trial) was 68.37 (SD: 62.42) m in the FES group and 57.50 (SD: 68.17) m in the control group (Table 2 and Table A1). There was no statistically significant difference between the groups (10.86 m; 95% CI: −8.26 to 29.98, *p* = 0.26).

In the secondary outcomes (Table 2), no statistically significant difference was observed between the groups other than the active dorsiflexion ROM, patient-reported outcome measures, and gait analysis (barefoot). Other outcomes of examinations with FES are presented in Appendix A.

There were seven (7%) cases and two (2%) cases of AEs in the FES and control groups, respectively (Table 3). The most frequent AE was falling (five events in three patients in the FES group), with mild severity in all cases, and no events were related to the trial device. One case of a serious AE occurred in the FES group (femur fracture, one event), whereas no such AEs were reported in the control group. In one patient in the FES group, the device malfunctioned due to Bluetooth and application failure, which was resolved through inspection and replacement of the device.

## 4. Discussion

To the best of our knowledge, this was the first large-scale randomized, controlled trial conducted to evaluate the effectiveness of FES in Japanese convalescent post-stroke patients with hemiplegic gait disorder. Previous studies with smaller sizes have shown that FES improves the quality of gait in non-Japanese patients with foot drop [7,8,9,10]. However, the effect of the FES device is expected to be different from that in Western patients because of the Japanese lifestyle, which is often spent barefoot, requiring complex muscle movements due to the instability of the ankle joint. In this trial, FES did not significantly improve the distance covered by post-stroke patients with foot drop in the barefoot 6-MWT, which was the primary outcome.

Only a few studies with small sample sizes (*n* < 30) have focused on convalescent stroke patients [21,22]. In our trial (*n* = 184), the mean duration of the convalescent period was 61.6 days. In general, considering the plasticity of the brain, the paralysis of lower limb function is best improved within 3 months after onset. As this trial focused on such a convalescent phase important for recovery from paralysis, it could offer valuable information. Previous studies have shown the effectiveness of FES in improving receptivity to gait [21] and improving ankle paralysis [22,23], but all of these studies were conducted with small numbers of patients. On the other hand, there are also reports that FES did not improve ankle paralysis [24], which may depend on the number of cases, the time since stroke onset, and the paralysis assessment scale. In the FES group in this study, there were no group differences in walking speed, cadence, or FAC grade, though there was a tendency toward improved receptivity to gait (Appendix A). The ROM for ankle dorsiflexion and Fugl–Meyer assessment were used to assess paralysis in our study. Although no improvement was observed with the Fugl–Meyer assessment, there was an improvement in the ankle dorsiflexion ROM. These findings suggest that the device selection, frequency of use, and study endpoint should be carefully considered in future studies.

As it is usual to walk indoors with bare feet in Japan, we chose the 6-MWT (barefoot) as the primary outcome measure for our trial. Wearing Walkaide^®^ enables patients to comfortably walk indoors with bare feet while feeling the ground with the soles of the foot. Everaert et al. [8] conducted a randomized, controlled, crossover trial with three parallel arms (*n* = 121) to compare the effectiveness of Walkaide^®^ and AFO in post-stroke patients within 1 year from onset (convalescent phase plus chronic phase, mean: 6.4 months; SD: 3.6 months). A large but non-significant difference in improvement was observed in walking speed (barefoot) when comparing Walkaide^®^ and AFO. Similarly, no significant difference was observed in either the 10-MWT (barefoot) or 6-MWT (barefoot) in our trial. In the trial of Everaert et al. [8], AFO was not used in the control group, which may have affected the difference. In many trials, FES was shown to be non-superior to AFO [10,25,26]. In particular, we speculated that allowing the control group to use AFO might have masked the effectiveness of FES in this study. In contrast, a significant difference was observed in the gait evaluation, when walking barefoot, by care providers and was better in the FES group. This suggests that FES can make patients walk correctly and cleanly, as reported by Sheffler et al. [22], although walking speed and distance were unchanged.

We analyzed the walking speed, stride length, gait, and walking distance within a specified time, as in the 6-MWT, 3-MWT, and 10-MWT, and observed significant improvements after the FES intervention in the 6-MWT (barefoot, FES, AFO), 10-MWT (barefoot, FES, AFO), and timed up and go test (degree of comfort in a maximum of barefoot, FES, AFO). However, as mentioned before, there is a possibility that the effectiveness of FES was unexpectedly masked because AFO use was allowed in the control group in our trial. Moreover, it was noteworthy that the barefoot gait evaluation in the stance phase, swing phase, and all phases showed a significant difference between the groups. In the previous reports, the evaluation methods were diverse, and the results varied depending on the equipment used for the intervention and the intervention methodology (frequency, duration, control, etc.). Generally, FES is not superior to AFO, and improvements with FES are similar to those with AFO in various walking evaluation methods. According to the report by Everaert et al. [8], significantly more patients preferred Walkaide^®^ as a supportive device after the study. The study by Salisbury et al. [21] also reported that the FES device tended to improve the patient’s receptivity to walking. Thus, patients experienced comfort while walking with the FES support, which did not change the walking speed or distance; this was reflected by the differences between the groups in the three patient-reported outcomes assessed. Post-stroke patients preferred Walkaide^®^ as a supportive device.

### Study Limitations

This study had some limitations. First, the inclusion criteria should have been broader, including patients with an earlier onset or lower walking ability. Second, more consideration should be given to the use of AFO in the control group to avoid affecting the results. Third, we should have promoted the selection of the TILT mode more actively because Walkaide^®^ is originally intended for use in the TILT mode. In future studies, these limitations should be considered when planning the study design. Lastly, gait disorders are complex in nature, involving not only weakness of the legs, but also spatio-temporal patterns, joint position sense [27], and other coordination disorders [28] that affect the nature of movement. Although we have tried to eliminate, as much as possible, the influence on gait, other than that of stroke, in the eligibility criteria, it is important to try to analyze in detail and stratify the data of the gait condition recordings conducted in this study.

## 5. Conclusions

FES did not significantly improve the distance covered during the barefoot 6-MWT performed by Japanese convalescent stroke patients with hemiplegic gait disorder (foot drop). A similar study design, the PLEASURE study [29] of chronic stroke patients, found that the magnitude of improvement in gait ability and ankle-specific body function in the FES group was similar to that in the control group, as did the results of this study. Electrical stimulation to promote ankle dorsiflexion with the WalkAide did not show efficacy in the treatment of Japanese convalescent stroke patients with drooping legs, but future work is needed to investigate the therapeutic effects of the device or stimulation conditions.

## Figures and Tables

**Table 1:** Baseline characteristics by treatment allocation. Data are presented as means (SDs) or *n* (%). Abbreviations: FAC, functional ambulation classification; MAS, modified Ashworth scale; SD, standard deviation.

		FES (*n* = 92)	Control (*n* = 92)
Age, years		63.5 (10.5)	64.3 (11.8)
Sex (male)		70 (76)	68 (74)
Body weight, kg		62.6 (10.9)	61.4 (12.0)
Time since stroke onset, days		59.5 (32.6)	63.7 (30.4)
Cause of hemiplegia	Cerebral hemorrhage	42 (46)	40 (43)
	Cerebral infarction	50 (54)	52 (57)
FAC category	3	49 (53)	52 (57)
	4	43 (47)	40 (43)
MAS score of plantar flexor muscles, knee extended	0	10 (11)	14 (15)
	1	28 (30)	27 (29)
	1+	32 (35)	31 (34)
	2	17 (18)	18 (20)
	3	5 (5)	1 (1)
	4	0 (0)	0 (0)
MAS score of plantar flexor muscles, knee flexed	0	21 (23)	21 (23)
	1	34 (37)	34 (37)
	1+	24 (26)	25 (27)
	2	11 (12)	10 (11)
	3	2 (2)	1 (1)
	4	0 (0)	0 (0)
Dorsiflexion range of motion	Active	5.5 (5.5)	7.6 (7.6)
	Passive, knee extended	7.0 (6.1)	6.0 (7.6)
	Passive, knee flexed	13.6 (6.6)	14.7 (7.5)

**Table 2:** Primary and secondary outcomes. Data are presented as means (SDs). No conclusions can be made regarding differences in secondary outcomes because of the lack of planned adjustment for multiple comparisons. Abbreviations: 6-MWT, 6 min walk test; AFO, ankle-foot orthosis; FMA, Fugl–Meyer Assessment.

		FES (*n* = 92)			Control (*n* = 92)			
		Baseline	Follow-Up	Change	Baseline	Follow-Up	Change	*p*-Value
6-MWT (barefoot) distance, m		164.21 (105.99)	232.57 (122.88)	68.37 (62.42)	153.87 (113.96)	211.37 (126.89)	57.50 (68.17)	0.26
6-MWT (with AFO) distance, m		179.46 (92.66)	238.24 (97.87)	58.78 (55.66)	178.61 (109.96)	245.00 (123.76)	66.40 (55.25)	0.39
10-m walk test (barefoot) speed, m/s		0.55 (0.29)	0.76 (0.31)	0.21 (0.18)	0.51 (0.3)	0.68 (0.37)	0.17 (0.17)	0.16
10-m walk test (with AFO) speed, m/s		0.54 (0.25)	0.71 (0.28)	0.17 (0.16)	0.55 (0.28)	0.71 (0.33)	0.16 (0.14)	0.86
Lower extremity FMA score		25.65 (4.87)	27.31 (4.33)	1.66 (2.49)	25.16 (5.14)	26.43 (5.29)	1.28 (2.9)	0.34
MAS score of plantar flexor muscles	Knee extended	1.36 (0.69)	1.21 (0.68)	−0.15 (0.67)	1.24 (0.66)	1.06 (0.67)	−0.18 (0.61)	0.76
	Knee flexed	1.06 (0.71)	1.07 (0.72)	0.01 (0.68)	1.04 (0.68)	0.90 (0.65)	−0.14 (0.65)	0.15
Dorsiflexion range of motion	Active	5.46 (5.57)	8.54 (6.54)	3.08 (4.21)	7.73 (7.6)	8.33 (8.7)	0.61 (4.25)	0.001
	Passive, knee flexed	13.62 (6.59)	14.66 (6.37)	1.03 (5.51)	14.72 (7.48)	15.06 (7.74)	0.34 (5.58)	0.41
Timed up and go test (barefoot), s	At comfortable speed	28.52 (15.88)	19.18 (10.09)	−9.34 (10.23)	31.29 (18.84)	22.84 (16.66)	−8.45 (10.71)	0.58
	At maximum speed	23.32 (13.07)	16.30 (9.82)	−7.03 (8.61)	27.02 (17.83)	18.99 (13.66)	−8.03 (10.24)	0.49
Timed up and go test (with AFO), s	At comfortable speed	27.81 (16.77)	20.12 (11.69)	−7.68 (12.45)	29.68 (19.66)	21.48 (15.65)	−8.20 (10.8)	0.78
	At maximum speed	23.32 (13.07)	16.30 (9.82)	−7.03 (8.61)	27.02 (17.83)	18.99 (13.66)	−8.03 (10.24)	0.49
Stroke Impact Scale	Mobility	53.49 (21.26)	76.17 (17.61)	22.69 (21.61)	52.17 (21.11)	71.71 (23.03)	19.54 (21.52)	0.33
	Total score	54.32 (12.17)	64.50 (13.84)	10.18 (11.92)	54.15 (13.32)	62.92 (14.3)	8.77 (10.9)	0.41
Patient-reported	Burden in raising the foot during barefoot walking	40.76 (22.23)	59.42 (20.57)	18.66 (23.44)	37.35 (23.73)	51.45 (22.76)	14.10 (24.32)	0.20
	Spasticity while walking bare-footed	50.53 (28.79)	67.58 (26.64)	17.05 (33.84)	57.49 (32.74)	60.08 (28.12)	2.59 (33.37)	0.005
	Stability in bare-footed walking	41.85 (24.93)	64.25 (23.22)	22.41 (22.69)	40.19 (25.36)	54.55 (26.79)	14.35 (24.91)	0.02
Gait disturbance evaluated by the care providers (barefoot)	At stance phase	3.15 (12.54)	9.38 (12.34)	6.23 (6.96)	1.99 (11.85)	6.05 (12.97)	4.06 (6.32)	0.04
	At swing phase	2.42 (10.23)	7.12 (10.26)	4.70 (5.45)	1.77 (9.79)	4.68 (10.53)	2.90 (4.71)	0.02
	At all phases	5.57 (22.69)	16.50 (22.52)	10.93 (12.17)	3.76 (21.55)	10.73 (23.44)	6.96 (10.79)	0.03
Gait disturbance (AFO)	At stance phase	2.76 (9.49)	8.28 (10.13)	5.53 (5.71)	2.79 (10.23)	6.87 (10.98)	4.08 (6.62)	0.15
	At swing phase	1.81 (7.99)	6.26 (8.26)	4.45 (4.38)	1.90 (8.48)	5.19 (9.02)	3.29 (5.09)	0.13
	At all phases	4.57 (17.37)	14.54 (18.31)	9.97 (9.81)	4.69 (18.63)	12.06 (19.94)	7.37 (11.53)	0.13

**Table 3:** Adverse events. Data are presented as *n* (%).

	FES (*n* = 94)	Control (*n* = 96)	*p*-Value
Any adverse event	7 (7)	2 (2)	0.10
Adverse events related to treatment	0	0	-
Any serious adverse event	1 (1)	0	0.49

## Data Availability

The data that support the findings of this study are available from the corresponding author, [S.M.], upon reasonable request.

## Appendix A

**Table A1:** Outcomes of examinations with FES. Data are presented as means (SDs). 6-MWT, 6 min walk test; FES, functional electrical stimulation.

		FES (*n* = 92)		
		Baseline	Follow-Up	Change
6-MWT distance, m		193.07 (108.05)	257.08 (110.47)	64.01 (61.99)
10 m walk test speed, m/s		0.58 (0.27)	0.78 (0.29)	0.20 (0.15)
Timed up and go test, s	At comfortable speed	25.78 (18.92)	18.18 (9.58)	−7.61 (16.8)
	At maximum speed	21.71 (18.12)	15.35 (8.12)	−6.37 (16.23)
Gait disturbance at 10 m walk test	At stance phase	6.27 (11.31)	11.83 (11.1)	5.56 (5.99)
	At swing phase	4.67 (9.24)	9.08 (8.91)	4.41 (4.69)
	At all phases	10.94 (20.48)	20.91 (19.96)	9.97 (10.51)

## Appendix B

**The RALLY trial investigators and committees**
**Principal Investigator:**
Shuji Matsumoto, Center of Medical Education, Faculty of Health Sciences, Ryotokuji University, Japan.
**Vice Principal Investigator:**
Megumi Shimodozono, Department of Rehabilitation and Physical Medicine, Graduate School of Medical and Dental Sciences, Kagoshima University, Japan.
Ryuji Miyata, Department of Rehabilitation and Physical Medicine, Graduate School of Medical and Dental Sciences, Kagoshima University, Japan.
Data And Safety Monitoring Committee.
Toyoko Asami, Department of Rehabilitation Medicine, Saga University Hospital, Japan.
Akihiko Oowatashi, School of Health Sciences, Faculty of Medicine, Kagoshima University, Japan.
Saburo Omine, Faculty of Rehabilitation, Kyusyu Nutrition Welfare University.
**Blinded Independent Central Review Board (Fugl–Meyer assessment, gait evaluation):**
Rina Ijichi, Department of Rehabilitation, Kirishima Sugiyasu Hospital, Japan.
Tetsuya Onoda, Department of Rehabilitation, Kirishima Medical Center, Japan.
**Trial Statistician:**
Masanori Taketsuna, Translational Research Center for Medical Innovation, Japan.
**Study sites and trial investigators:**
Jun Ohkawara, Department of Rehabilitation, Ohkawara Neurosurgical Hospital.
Takashi Shigematsu, Shintaro Iio, Tetsuya Suzuki, Department of Rehabilitation, Hamamatsu City Rehabilitation Hospital.
Akira Satone, Keisuke Ono, Senshuu Abe, Eri Tanita, Department of Rehabilitation, Tokachi Rehabilitation Center.
Hidenobu Okuma, Hironori Fujisaki, Department of Rehabilitation, Kumamoto Takumadai Rehabilitation Hospital.
Makiko Seto, Junya Sasahara, Hiroyuki Yamamoto, Department of Rehabilitation, Nagasaki Kita Hospital.
Shigeatsu Natsume, Masamori Fujiwara, Department of Rehabilitation, Eishokai Medical Corporation, Yoshida Hospital Cerebrovascular Research Institute.
Norihito Kimura, Department of Rehabilitation, Caress Sapporo Tokeidai Memorial Hospital.
Hirokazu Kawano, Department of Rehabilitation, Junwakai Memorial Hospital.
Osamu Kira, Yousuke Miyanaga, Department of Rehabilitation Therapy, Junwakai Memorial Hospital.
Kenji Matsumoto, Makiko Hayakawa, Department of Rehabilitation, Kansai Rehabilitation Hospital.
Yuji Hashimoto, Department of Rehabilitation, Sapporo Shiroishi Memorial Hospital.
Yasuhide Kido, Osamu Yamazaki, Yuuichi Suzuki, Department of Rehabilitation, Matsuyama Rehabilitation Hospital.
Megumi Shimodozono, Rintaro Ohama, Ryuji Miyata, Department of Rehabilitation and Physical Medicine, Graduate School of Medical and Dental Sciences, Kagoshima University.
Tomohiro Uema, Department of Rehabilitation, Kagoshima University Hospital.
Makoto Ide, Koichiro Tobinaga, Tsutomu Asou, Shun Kumamoto, Department of Rehabilitation, St Mary’s Healthcare Center.
Keizo Shigenobu, Yumeko Amano, Department of Rehabilitation, Kohshinkai Ogura Hospital.
Toshihiro Nakamura, Kota Homan, Department of Rehabilitation, Acras Central Hospital.
Yuki Onishi, Atsushi Manji, Kazuki Ogashira, Department of Rehabilitation, Saitama Misato General Rehabilitation Hospital.
Tojiro Yanagi, Tetuya Noda, Horoki Fukuda, Department of Rehabilitation, Yame Rehabili Hospital.
Katsuhiro Harada, Yuki Nakama, Keisuke Shibuya, Department of Rehabilitation, Fujimoto Kamimachi Hospital.
Kanjiro Suzuki, Nobuaki Oshikawa, Tatsuya Yamashita, Department of Rehabilitation, Nichinan Municipal Chubu Hospital.
You Ikegami, Kouhei Totiki, Department of Rehabilitation, Yohkoh Rehabilitation Hospital.
Taisuke Arai, Kenji Ogushi, Shinya Nakagawa, Department of Rehabilitation, Shin Yachiyo Hospital.
Rintaro Ohama, Yuji Sakashita, Kazutoshi Tomioka, Sota Araki, Department of Rehabilitation, Tarumizu Municipal Medical Center, Tarumizu Central Hospital.
Yuko Takiyoshi, Hirohisa Oyadomari, Wakako Omine, Sayoko Hirayama, Department of Rehabilitation, Nambu Tokushukai Hospital.
Shoichi Tanaka, Keisuke Sato, Takafumi Miyagi, Department of Rehabilitation, Chuzan Hospital.
Masahiko Toshima, Kazuhiro Yanai, Naoki Shoji, Yuto Yamamoto. Department of Rehabilitation, Hokusei Memorial Hospital. Makoto Kawasaki, Sakurajyuji Hospital.
Satoru Matayoshi, Department of Rehabilitation, Okinawa Rehabilitation Center Hospital.
Keisuke Horinouchi, Department of Rehabilitation, Kajikionsen Hospital.
Yuichi Komaba, Daiki Ooshima, Department of Rehabilitation, Hakujikai Memorial Hospital.
Taro Ogawa, Department of Rehabilitation, Sapporo Keijinkai Rehabilitation Hospital.
Jun Takeshita, Department of Rehabilitation, IMS Itabashi Rehabilitation Hospital.
**Data Center:**
Translational Research Center for Medical Innovation.
Satoshi Nakagawa, Project Manager.
Kuniko Hosokawa, Data Manager.
Yoko Nakagawa, Statistical Programmer.
